# Supplementary material for: Multiwalled Carbon Nanotubes Embedded in a Polymeric Matrix as a New Material for Thin Film Microextraction (TFME) in Organic Pollutant Monitoring
Source: Polymers (Basel). 2023 Jan 7;15(2):314. doi: 10.3390/polym15020314 (PMC9863860; doi:10.3390/polym15020314)
Supplement: Supplementary file 1 [file polymers-15-00314-s001.zip › polymers-2102282-supplementary.pdf]

# Supplementary Materials

**Multiwalled carbon nanotubes embedded in a polymeric matrix as a new material for thin film microextraction (TFME) in organic pollutant monitoring**

Ivonne Quintanilla[1], Carlos Perelló[1], Francesca Merlo[2], Antonella Profumo[2], Claudia Fontàs[1], Enriqueta Anticó[1]
[1]*Department of Chemistry, Universitat de Girona, C/ Maria Aurèlia Capmany 69, 17003 Girona, Spain.*
[2]*Department of Chemistry, University of Pavia, Via Taramelli 12, 27100 Pavia, Italy.*

**Preliminary results on the extraction of CPS and AHTN with films containing 5% MWCNTs and 10% MWCNTs**

The procedure was adapted from reference 16. Briefly, a 20 mL solution containing 50 µg $L^{-1}$ CPS and AHTN were placed in contact under rotary agitation with a piece of film (4 $cm^2$) for 6 h. The % extraction was calculated from the peak area at t= 6 h in relation to the peak area at t= 0.

**MWCNT characteristics**

XRD spectrum is shown below.

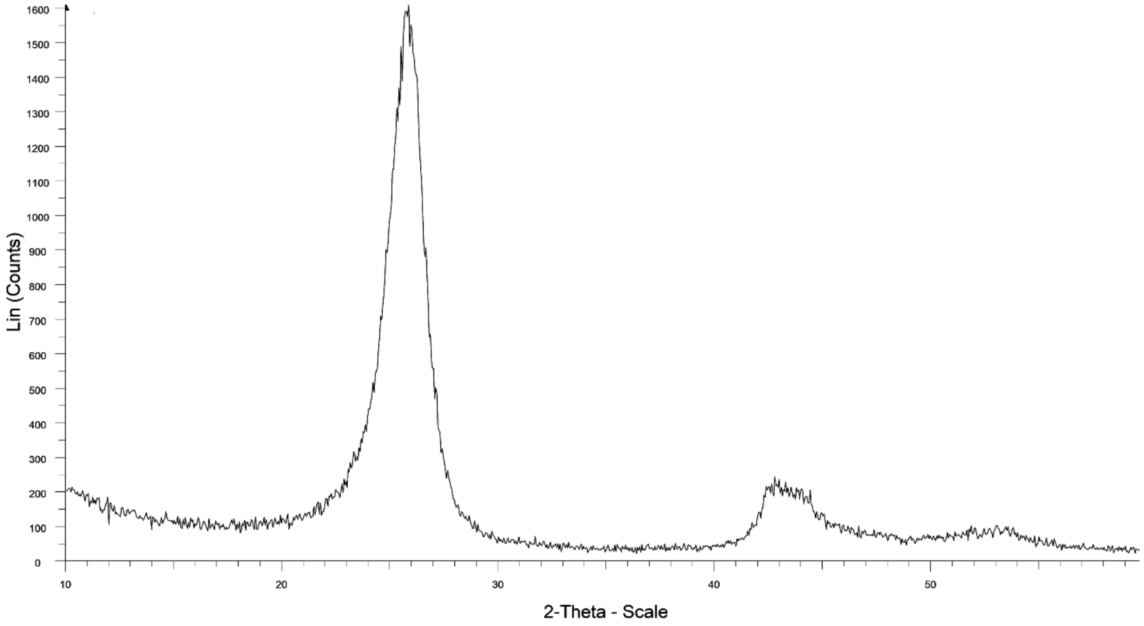


**Figure S1**. XRD spectrum of MWCNTs from Sigma Aldrich.

Measurement was performed on a Bruker D8 ADVANCE diffractometer with Bragg-Brentano Theta – 2Theta geometry, reflection mode using Cu Kα radiation (λ = 1.5406 Å) from an X-Ray tube. A secondary graphite monochromator was used. The experimental XRD pattern matches the ones found in the literature, with the strong peak of the (002) reflection at 25.81 degrees (2Theta).

According to the details supplied by the vendor, these arc-discharge multiwall carbon nanotubes (MWNTs) are catalyst free. They consist of a multi-walled carbon nanotube core surrounded by a fused carbon shell, the remainder being made up of multi-layer polygonal carbon nanoparticles and amorphous and graphitic carbon nanoparticles. It contains approximately 5-20 graphitic layers.
